# Protection of pulmonary epithelial cells from oxidative stress by *hMYH *adenine glycosylase

**DOI:** 10.1186/1465-9921-5-16

**Published:** 2004-09-27

**Authors:** Ted M Kremer, Mikael L Rinne, Yi Xu, Xian Ming Chen, Mark R Kelley

**Affiliations:** 1 Department of Pediatrics, Herman B Wells Center for Pediatric Research, Indiana University School of Medicine, Indianapolis, Indiana, USA

## Abstract

**Background:**

Oxygen toxicity is a major cause of lung injury. The base excision repair pathway is one of the most important cellular protection mechanisms that responds to oxidative DNA damage. Lesion-specific DNA repair enzymes include *hOgg1*, *hMYH*, *hNTH *and *hMTH.*

**Methods:**

The above lesion-specific DNA repair enzymes were expressed in human alveolar epithelial cells (A549) using the pSF91.1 retroviral vector. Cells were exposed to a 95% oxygen environment, ionizing radiation (IR), or H_2_O_2_. Cell growth analysis was performed under non-toxic conditions. Western blot analysis was performed to verify over-expression and assess endogenous expression under toxic and non-toxic conditions. Statistical analysis was performed using the paired Student's *t *test with significance being accepted for p < 0.05.

**Results:**

Cell killing assays demonstrated cells over-expressing *hMYH *had improved survival to both increased oxygen and IR. Cell growth analysis of A549 cells under non-toxic conditions revealed cells over-expressing *hMYH *also grow at a slower rate. Western blot analysis demonstrated over-expression of each individual gene and did not result in altered endogenous expression of the others. However, it was observed that O_2 _toxicity did lead to a reduced endogenous expression of *hNTH *in A549 cells.

**Conclusion:**

Increased expression of the DNA glycosylase repair enzyme *hMYH *in A549 cells exposed to O_2 _and IR leads to improvements in cell survival. DNA repair through the base excision repair pathway may provide an alternative way to offset the damaging effects of O_2 _and its metabolites.

## Background

Oxidative stress leading to the overproduction of free radicals in the lungs is present in many clinical situations. Such clinical settings include acute respiratory distress syndrome (ARDS), infants of prematurity going on to develop bronchopulmonary dysplasia (BPD), pathogenesis of chronic obstructive pulmonary disease (COPD), asthma, cystic fibrosis, ischemia-reperfusion injury, drug-induced lung toxicity, cancer and aging [1-4]. Although the use of oxygen may be clinically indicated in hypoxemic situations, one must consider the potential long-term toxic side effects. For example, we know that oxygen creates cellular damage by a variety of mechanisms. Normal cellular metabolism of oxygen involves the transfer of electrons from NADH to O_2 _molecules to form water (H_2_O). At normal partial pressure, 95% of oxygen molecules (O_2_) are reduced to H_2_O and 5% are partially reduced to toxic byproducts by normal metabolism in the mitochondria [5]. These metabolites include the superoxide anion (O_2_^-^), hydrogen peroxide (H_2_O_2_), and hydroxyl radicals (^•^OH) all of which make up what are known as Reactive Oxygen Species (ROS) [6]. Exposure to conditions of hyperoxia as well as ionizing radiation (IR) leads to increased amounts of these ROS and their damaging effects.

ROS are known to attack the lipids, proteins, and nucleic acids of cells and tissues [5,7]. Lipids, including pulmonary surfactant, react with ROS to produce lipid peroxides, which cause increased membrane permeability, inactivation of surfactant, and inhibition of normal cellular enzyme processes. Proteins reacting with ROS result in decreased protein synthesis due to inhibition of ribosomal translation or destruction of formed proteins. This ultimately leads to inactivation of intracellular enzymes and transport proteins resulting in impaired cellular metabolism and accumulation of cellular waste products. Lastly, ROS cause damage to nucleic acids by leading to modified purine and pyrimidine bases, apurinic (AP) /apyrimidinic sites, and DNA protein cross-links which can lead to single strand breaks [8].

Several defense mechanisms exist to combat the damaging effects of ROS. Intracellular enzymatic systems include superoxide dismutase which eliminates the superoxide anion, catalase which catalyzes the reduction of H_2_O_2 _directly to H_2_O without the production of the hydroxyl radical, and glutathione peroxidase which directly reduces H_2_O_2 _and lipid peroxides. Free radical scavengers, which stop free radical chain reactions by accepting electrons, include α-tocopheral (vitamin E), ascorbic acid (vitamin C), niacin (vitamin B), riboflavin (vitamin B_2_), vitamin A, and ceruloplasmin [1,2,9]. These systems usually provide enough protection against oxygen metabolism under normal conditions, but may become depleted under conditions of increased oxidative stress [7,10].

**Figure 1:**
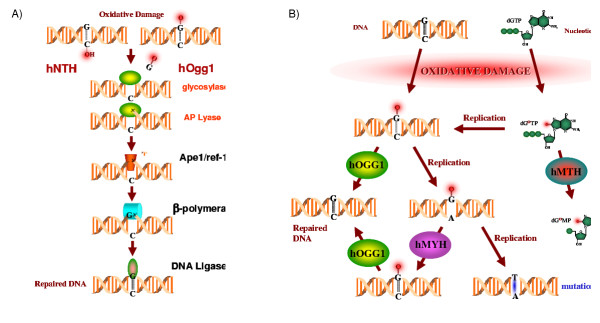
**Base excision repair pathways for Oxidative DNA damage. **(A) BER pathway demonstrating repair of 8-oxoG by the repair enzymes *hOgg1 *and *hNTH*. (B) *hOgg1*, *hMYH*, and *hMTH *and their respective repair function.

The defense mechanism of interest in this paper involves the repair of oxidative damage through the human DNA base excision repair pathway (BER). BER is the most important cellular protection mechanism that removes oxidative DNA damage [11]. Damaged bases are excised and replaced in a multi-step process. Lesion-specific DNA glycosylase repair genes initiate this process. After removal of the damaged base, the resulting AP site is cleaved by AP-endonuclease generating a 3'OH and 5'deoxyribose phosphate (dRP). β-polymerase, which possesses dRPase activity, cleaves the dRP residue generating a nucleotide gap and then fills in this single nucleotide gap. The final nick is sealed by DNA ligase [12-14] (Figure 1A).

The oxidative repair genes that we have analyzed in this study include 8-oxoguanine DNA glycosylase (*hOgg1*), human Mut Y homologue (*hMYH*), human Mut T homologue (*hMTH*), and endonuclease III (*hNTH*) all of which are present in human cells and involved in the protection of DNA from oxidative damage. The repair enzyme *hOgg1 *is a purine oxidation glycosylase that recognizes and excise 8-oxoguanine lesions (GO) paired with cytosine. GO can pair with both cytosine and adenine during DNA replication [15]. If repair of C/GO does not occur, then G:C to T:A transversions may result [5,15-17]. The repair enzyme *hMYH *is an 8-oxoguanine mismatch glycosylase that removes adenines misincorporated opposite 8-oxoG lesions that arise through DNA replication errors [5,18-20]. The repair enzyme *hMTH *hydrolyzes oxidized purine nucleoside triphosphates such as 8-oxo-dGTP, 8-oxo-GTP, 8-oxo-dATP, and 2-hydroxy-dATP, effectively removing them from the nucleotide pool and preventing their incorporation into DNA (Figure 1B) [21]. Lastly, the repair gene endonuclease III (*hNTH*) is a pyrimidine oxidation and hydration glycosylase that recognizes a wide range of damaged pyrimidines [22]. *hNTH *has also been shown to have a similar DNA glycosylase/AP lyase activity that can remove 8-oxoG from 8-oxoG/G, 8-oxoG/A, and 8-oxoG/C mispairs [23,24]. Subsequent steps following *hNTH *are identical to those following *hOgg1 *(Figure 1A).

A previous study has shown that over-expression of the DNA repair gene *hOgg1 *leads to reduced hyperoxia-induced DNA damage in human alveolar epithelial cells [25]. The primary goal of our present study was to compare the protective effects of the four main lesion-specific DNA glycosylase repair genes by individually over-expressing each in lung cells and determining which of these provides the greatest degree of protection under conditions of increased oxidative stress.

## Methods

### Cell Culture

The human alveolar epithelial cell line A549 (58 year old Caucasian male), was purchased from ATCC Cat No CCL-185. The cells were grown in DMEM (Gibco, Grand Island, NY) supplemented with 10% fetal bovine serum (FBS) (HyClone, Logan, UT) and penicillin (100 U/ml)/streptomycin (100 μg/ml) (Gibco, Grand Island, NY). Passaging of cells was performed every 3–4 days with cells grown to 80% confluency in a 10 cm cell culture dish (Corning Incorporated, Corning, NY). Cells were kept at 37°C in a humidified, 5% CO2 incubator.

### Retroviral Vector Construction

The retroviral vector pSF91.1, a gift from Dr. C. Baum from the University of Hamburg in Germany, was constructed with an internal ribosome entry site (IRES) upstream to the gene expressing enhanced green fluorescent protein (EGFP) as previously described [26].

Four DNA repair genes were individually ligated into the retroviral vector pSF91.1.

#### hOgg1-6pcDNA3.1

was initially amplified by PCR by primers to introduce a kozak sequence at the 5' end [27]. Digestion of this product with EcoRI and SalI was performed and then *hOgg1 *was subcloned into digested plasmid vector pSF91.1, with T4 DNA ligase. DNA sequencing was performed to confirm integrity of the *hOgg1 *gene.

#### hMYH/PGEX4T-1 and hMTH/PGEX4T-1

*hMYH *was a gift from Dr. A. McCullough (University of Texas Medical School, Galveston, TX) and *hMTH *was cloned in Dr. Kelley's lab. Plasmid DNA was prepared as above by digestion with EcoRI and SalI and ligated into pSF91.1 as above and sequencing was performed to confirm integrity of the genes.

#### PGEX-6PI-hNTH1-wild type

this gene was a gift from Dr. S. Mitra (University of Texas Medical School, Galveston, TX). Digestion with BamHI and SalI was performed and the *hNTH1-wt *fragment was ligated into the empty plasmid vector PUC18. The *hNTH1-wt *fragment was then excised with both sides flanked by EcoRI restriction sites and ligated into pSF91.1. Proper orientation of the gene was confirmed and sequencing was performed to determine the integrity of the gene.

### Retroviral Production and Infection

DH5α competent cells (Life Technologies, Gaithersburg, MD) with each of the five DNA repair genes were grown in LB-broth with ampicillin (Sigma, St. Louis, MO). Plasmid DNA was prepared and used to transfect phoenix amphotropic cells, from the Nolan Lab (Stanford University Medical Center, San Francisco, CA), grown to ~80% confluency. On the second day sodium butyrate was added to each plate and incubated at 37°C for 6 hours. Fresh DMEM supplemented with FBS and penicillin/streptomycin was added and the plates were incubated at 33°C. Viral supernatant was collected 24 and 48 hrs later, filtered through a 0.45 μm acrodisc syringe filter (Pall Corporation, Ann Arbor, MI) and frozen at -80°C for later use. Retroviral titers were determined by fluorescent-activated cell sorter (FACS) analysis. Titers of viral supernatant were 8 × 10^5 ^to 1.2 × 10^6 ^particles/ml [26].

2.5 × 10^5 ^A549 cells were suspended with the viral supernatant and plated in 1 well of a 6-well plate along with polybrene (Sigma, St. Louis, MO). This exposure was performed 6 hours per day for three days. At approximately five days from the beginning of the infection, the infected cells were analyzed using flow cytometry and sorted for EGFP expression.

### Western Analysis

Cell pellets of sorted cells were resuspended in NuPage buffer (Invitrogen, Carlsbad, CA) and protein concentrations were determined using the DC protein assay (Bio-Rad, Hercules, CA). 20 ug of protein were loaded into individual lanes of a NuPage Bis-Tris Gel (Invitrogen, Carlsbad, CA). The gel was then transferred to nitrocellulose paper (Osmonics Inc, Gloucester, MA). The membranes were then blocked with 1% blocking solution (Roche Diagnostics, Indianapolis, IN) for 1 hour at room temperature and then incubated overnight at 4°C with rabbit polyclonal antibodies to *hOgg1 *(Novus Biologicals, Littleton, CO), *hMTH *(Novus Biologicals, Littleton, CO), *hMYH *(Oncogene Research Products, Darmstadt, Germany) and *hNTH *(Proteintech Group Inc, Chicago, IL) all at a dilution of 1:1000 except *hNTH *which was diluted 1:2500. They were then washed 2 times with TBST and 2 times with 0.5% blocking solution, 10 minutes per wash. The membranes were incubated with anti-rabbit secondary antibodies at 1:1000 for 1 hour at room temperature. Lastly, the membranes were washed 4 times with TBST, 15 minutes per wash. The membranes were briefly soaked in BM chemiluminescence blotting substrate (Roche Diagnostics, Indianapolis, IN) and then exposed to high performance autoradiography film (Amersham Biosciences, Buckinghamshire, England). Kodak Digital Science 1D Image Analysis software was utilized to quantify the region of interest (ROI) band mass of individual bands on films where visualized differences were detected.

### Hyperoxic Exposure

Sorted EGFP positive A549 cells infected with the above DNA repair genes were counted and seeded into 96-well plates at a density of 1000 cells/well, 6 wells per gene. Six hours after seeding, individual plates were placed into an oxygen chamber supplied by Dr. L. Haneline (Wells Center for Research, Indianapolis, IN) located in a 37°C incubator. The oxygen chamber was then infused with 95% O_2 _and 5% CO_2_. Individual plates were removed after 12, 24, 48, and 72 hours of exposure. Control A549 cells were incubated in a normal 37°C humidified-5% CO_2 _incubator. O_2 _concentrations were monitored with a MAXO_2 _analyzer (Maxtec, Salt Lake City, UT). Four days from the beginning of the exposure, cells were assessed for cell growth/survival using the sulforhodamine B assay (SRB assay).

### Sulforhodamine B Assay

The SRB assay (Sigma, St. Louis, MO), developed by the National Cancer Institute, provides a sensitive measure of drug-induced cytotoxicity through a colorimetric endpoint that is non-destructive, indefinitely stable, and visible to the naked eye. This assay was used to assess the cell growth/survival of over-expressed cells [28]. Cold 10% TCA was used to fix the cells to the plate. After incubation for 1 hour at 4°C, the individual wells were rinsed with water. After air-drying, SRB solution was added to each well and cells were allowed to stain for 20–30 minutes. 1% acetic acid wash was used to rinse off unincorporated dye. Incorporated dye was then solubilized in 100 μl per well of 10 mM Tris. Absorbance was measured by a tunable microplate reader (Molecular Devices, Sunnyvale, CA) at a wavelength of 565 nm. Background absorbance measured at 690 nm was subtracted from the measurements at 565 nm.

### Irradiation and H_2_O_2 _Exposure

Sorted EGFP positive A549 cells were seeded into 96-well plates at a density of 1000 cells/well. Six hours after seeding, individual plates were then exposed to radiation at doses of 250, 500, 1000, and 1500 Rads or 0.2 mM, 0.4 mM, and 0.6 mM H_2_O_2 _(Sigma, St. Louis, MO). All plates including control plates were then placed into a 37°C humidified-5% CO_2 _incubator. Four days after exposure, cells were fixed and assessed for cell growth/survival by the SRB assay.

### Natural Cell growth

Sorted EGFP positive A549 cells and wild type cells were seeded individually onto four 96-well plates at 1000 cells/well. All the plates were placed into a 37°C humidified-5% CO_2 _incubator. Every 24 hours for 4 days, 1 plate was removed and the cells were fixed and analyzed by the SRB assay looking at cell growth under non-toxic conditions. Growth curves and exponential growth equations were determined to look at the doubling time (DT) of cells infected with each repair gene of interest compared to vector infected and uninfected wild type cells.

### Statistics

All drug exposure experiments were performed at least three times and individual drug doses included 6–8 wells for each group of infected cells. Analysis of cell growth and exponential growth equations were determined using Microsoft Excel. All experiments involving drug exposures were normalized to the zero dose. Data are expressed as means ± SE. The significance of differences were calculated using the paired Student's *t *test with significance being accepted for p < 0.05.

## Results

### Retroviral Constructs

**Figure 2:**
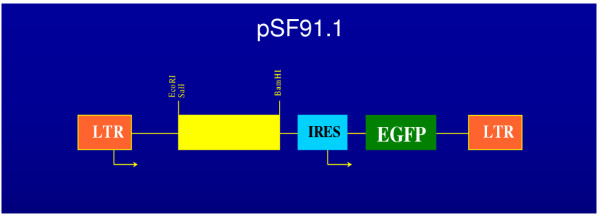
**Retroviral vector pSF91.1. **Depiction of the retroviral vector utilized in these experiments demonstrating restriction sites and location of entry of the gene of interest between the LTR and the IRES.

The DNA repair genes *hOgg1*, *hMYH*, *hMTH*, and *hNTH *were ligated into the retroviral vector pSF91.1 (figure 2). This vector, derived from a murine stem cell virus backbone, along with each individual repair gene, was used for transfection of phoenix amphotropic cells. Viral supernatant was then collected and used to stably infect A549 cells. A heterogeneous population of A549 cells expressing EGFP was sorted so all cells used for experiments contained the genes of interest integrated into their DNA (data not shown).

### Repair Gene Expression

Western blot analysis was performed on sorted cells in order to verify over-expression of the four genes of interest. *hOgg1*, *hMYH*, *hMTH*, and *hNTH *were all detected at their correct position on western blots (data not shown).

**Figure 3:**
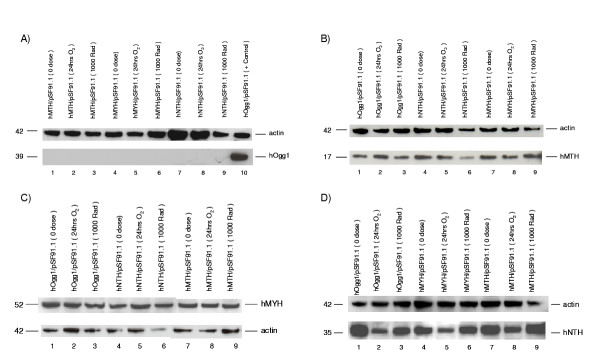
**Western analysis of A549 cells over-expressing individual repair genes and effect on endogenous glycosylase level. **(A) Endogenous expression of hOgg1 was not altered in A549 cells over-expressing any of the other repair genes when analyzed after non-toxic and toxic exposures. *hOgg1 *protein was not detectable for any of the cells under the above conditions when compared to cells over expressing *hOgg1*. (B) and (C) Endogenous expression of hMTH and hMYH respectively also were not altered in A549 cells over-expressing any of the other repair genes when analyzed after non-toxic and toxic exposures. (D) Endogenous expression of hNTH was analyzed under non-toxic and toxic conditions in A549 cells over-expressing the other repair genes. Reduced expression of hNTH was observed equally with all of the other genes after exposure to 95% O_2_. Endogenous expression of all four genes was equivalent under the above conditions in vector control cells; pSF91.1 (data not shown).

Western analysis was also utilized to assess whether over-expression of each individual repair gene resulted in altered endogenous expression of the other repair genes under both non-toxic and toxic conditions (24 hrs of 95% O_2 _and 1000 Rad). Cells over-expressing the repair genes *hOgg1*, *hMYH*, *hMTH*, and *hNTH *did not lead to altered expression of the other endogenous repair genes under the above conditions when compared to each other or pSF91.1 vector control cells (Figure 3A,3B,3C and 3D). *hOgg1*'s endogenous expression was below the level of detection. The pattern of endogenous expression of *hNTH *was consistent for each condition when comparing cells over-expressing *hOgg1*, *hMYH*, *hMTH*, and pSF91.1. Reduced expression of *hNTH *after exposure to 95% O_2 _was noted.

**Figure 4:**
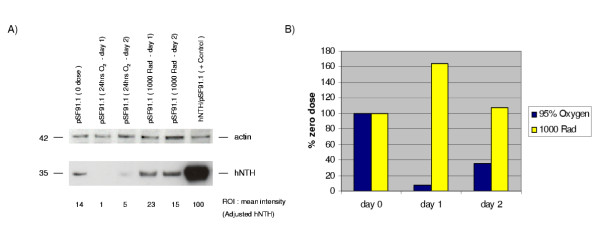
**Western analysis of endogenous *hNTH *repair gene after exposure to O_2 _and IR. **(A) Analysis of *hNTH *expression in A549 vector control cells following O_2 _or IR treatment. The ROI band mass mean intensity was calculated for individual bands and *hNTH *expression was normalized to the corresponding actin loading control. (B) Graph of ROI band mass normalized to the pSF91.1 zero dose.

Lastly, we assessed endogenous expression of each individual repair gene in cells infected with pSF91.1 following non-toxic and toxic conditions (24 hrs of 95% O_2 _and 1000 Rad) at 24 and 48 hrs after the onset of exposure. Endogenous *hMYH *and *hMTH *were expressed to the same degree. *hOgg1*'s endogenous expression was below the level of detection using western analysis (results not shown). When analyzing endogenous *hNTH *expression, it was noted that hyperoxia at 24 hrs and 48 hrs resulted in reduced protein expression by 93% and 64% respectively. There also was a small increase in expression of *hNTH *noted after 1000 Rad one day post exposure that was back to baseline by two days post exposure. ROI band mass quantification demonstrated this finding (Figure 4A and 4B). Two or more replicates were performed for each western analysis to determine consistency of the results.

### Protection from Hyperoxia and Radiation

**Figure 5:**
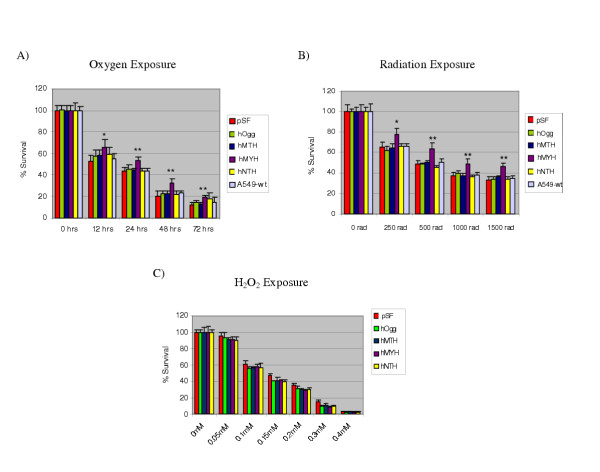
**Cell survival analysis following O_2_, IR, and H_2_O_2 _treatments. **A549 cells over-expressing *hOgg1*, *hMYH*, *hMTH *and *hNTH *following (A) O_2_, (B) IR, and (C) H_2_O_2_. Brackets indicate statistical significance at * p < 0.05 and ** p < 0.001 compared to pSF91.1 at each individual dose for 1 representative experiment.

A549 cells expressing *hMYH *demonstrated increased survival after exposure to conditions with elevated levels of oxygen compared to cells expressing only the pSF91.1 vector (Figure 5A). Results were highly significant at all time points except after 12 hours O_2 _where it almost reached a highly significant value. The differences between pSF91.1 and *hMYH *varied from 12% after 12 hours O_2 _exposure to 7% after 72 hours O_2 _exposure. A549 cells expressing *hMYH *also demonstrated increased survival after exposure to all doses of radiation in comparison to pSF91.1 (Figure 5B). These results were also highly significant at all doses of radiation except at 250 Rads where it almost reached a highly significant value. The differences between pSF91.1 and *hMYH *varied from 12%–14% for all doses of radiation. Also noted in these experiments was that vector control cells demonstrated no significant difference in survival at all doses of O_2 _and radiation in comparison to wild type A549 cells.

Experiments looking at the effects of H_2_O_2 _on cells expressing the repair genes did not demonstrate increased survival for any of these repair genes when compared to vector control cells (Figure 5C). This data demonstrates that over-expression of *hMYH *has the ability to improve cellular survival under conditions of hyperoxia and radiation but may not be able to overcome the toxicity of H_2_O_2_.

### Cell Growth

**Figure 6:**
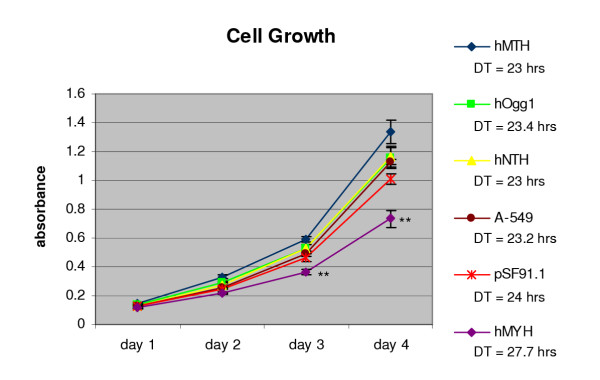
**Cell growth curve and associated doubling times (DT). **A549 cells over-expressing *hMYH *grow at a slower rate in comparison to all other cells under non-toxic conditions resulting in a prolongation of the doubling time. Of note, all other over-expressed cells have approximately the same doubling time as wild type A549 cells. Statistical significance noted at ** p < 0.001 compared to pSF91.1 for 1 representative experiment.

Cell growth under normal conditions was ascertained to determine if over-expression of any of the repair genes caused an alteration in the growth of cells in the absence of oxidative stress. Wild type A549 cells and cells expressing pSF91.1, *hNTH*, *hOgg1*, and *hMTH *appeared to grow at similar rates with doubling times within the same range. A549 cells expressing *hMYH *did show a slower growth rate that resulted in significant differences in cell number by day 3. The calculated doubling time for the cells over expressing *hMYH *is > 3 hrs longer than the cells with the other repair genes and vector alone (Figure 6). This slowing of growth may allow for more time to repair DNA damage, ultimately leading to increased cell survival following oxidative stress.

## Discussion

Oxidative stress to the lung leads to cellular DNA damage as evidenced by the release of specific gene products known to regulate DNA base excision repair pathways such as p53 and p21 [29-31]. Alterations in pro-inflammatory mediators, transcription factors, and other related gene products are also observed [32]. This injury has been shown to be associated with features of both cellular necrosis and apoptosis [33-35]. The resultant cellular inflammation and death from oxidative stress has a dramatic impact on the outcome of patients in the clinical setting [7,36].

Most of our current clinical therapy towards oxidative stress in the lung involves both supportive measures and prevention. Research dealing with oxidative lung injury has focused mainly on enhancing antioxidant enzymatic processes and free radical scavengers [37-40]. The ability to alter cellular survival by increasing specific DNA repair mechanisms may add another approach to the treatment of oxidant-mediated lung injury.

Many investigators have used hydrogen peroxide as a substitute for hyperoxia since it is known to be one of the metabolites produced by the metabolism of oxygen. ROS such as H_2_O_2 _and those produced by hyperoxia clearly lead to DNA damage but questions exist as to whether H_2_O_2 _leads to the same deleterious effects upon DNA as hyperoxia. Analysis of our growth curves after exposure to H_2_O_2 _in comparison to hyperoxia and IR clearly indicates that cellular protection by oxidative DNA repair genes is specific to the agent used. Because no protection was observed with over-expression of any of the repair genes following exposure to H_2_O_2_, we speculate that the damage it causes is dissimilar. It may be that its damage not only involves oxidized bases, but may also include other forms of DNA, lipid, and protein damage that are not corrected by oxidative DNA repair genes. Alternatively, the amount and type of damage evoked by H_2_O_2 _could be beyond that which can be corrected by over-expressing these repair genes.

Another form of stress known to induce damage through the formation of ROS is IR. Radiation induced free radical damage to DNA has substantial overlap with that of oxidative damage [41-43]. The protection provided by specific oxidative DNA repair genes under conditions of IR, was notable throughout our experiments only with the repair enzyme *hMYH*.

The primary agent utilized to induce the formation of ROS was an oxygen rich environment. The use of oxygen as a stressor leading to the formation of ROS, offers a distinct advantage over IR and H_2_O_2 _by mimicking the clinical situation where constant exposure to hyperoxia leads to cumulative cellular damage which further compromises repair. We determined that survival of A549 cells was also enhanced to a small degree with increased expression of the repair enzyme *hMYH*. This was an unexpected finding as we anticipated the repair gene *hOgg1 *would demonstrate the greatest protection in response to oxidative stress based on previous studies, however these experiments utilized the colony forming assay (CFA) to detect improvements in survival [25]. Additionally, the CFA may provide different results compared to the SRB assay, which allows for growth analysis over a shorter window of time. Furthermore, their study did not look at the repair enzyme *hMYH *and its impact on survival. Another study has investigated the repair function of *hMYH *in MYH-deficient murine cells. It was demonstrated that transfection of the MYH-deficient cells with a wild-type MYH expression vector increased the efficiency of A:GO repair [44].

An interesting observation noted while doing our experiments lead us to look at individual growth characteristics of cells over-expressing each of the oxidative repair enzymes. Cells over-expressing the repair enzyme *hMYH *clearly grow at a slower rate when compared with the other enzymes. The mechanism behind this is not understood at this point in time. The repair action of *hMYH *is known to remove adenines misincorporated opposite 8-oxoG lesions. This lesion occurs when a C/GO lesion is allowed to replicate before being corrected by *hOgg1*. Repair by *hMYH *is not a final corrective measure. The product of *hMYH *activity is the lesion C/GO, which allows *hOgg1 *to have another opportunity to remove 8-oxoG opposite cytosine. We know that A549 cells possess the *hOgg1 *gene based on a previous study demonstrating the presence of this gene after amplification by genomic PCR [45]. We also have demonstrated endogenous activity of *hOgg1 *in A549 cells by using an 8-oxoguanine bioactivity assay. Therefore, our explanation of these results is that the slowed growth created by *hMYH *may provide a wider window of opportunity for the repair process to take place, which ultimately grants endogenous *hOgg1 *another opportunity to remove the 8-oxoG lesion created by oxidative stress.

As noted in the methods section, the SRB assay provides a sensitive measure of drug-induced cytotoxicity that is used to assess cell proliferation/survival. The reduced cell proliferation of A549 cells over-expressing hMYH under non-toxic conditions may likely underestimate the magnitude of the protective effect of this particular repair enzyme. This may in fact make the results even more significant.

Recent studies have discovered hereditary variations of the glycosylase *hMYH *that may predispose to familial colorectal cancer [46,47]. Others have looked for *hMYH *variants in lung cancer patients and have not identified any clear pathogenic biallelic *hMYH *mutations or an over-representation of *hMYH *polymorphisms [47]. The A549 cell line has not demonstrated somatic mutations in *hMYH*, but a single nucleotide polymorphism (SNPs) has been noted [45]. The impact on function by this SNP is unknown. It would appear that the function of *hMYH *is very important in preventing somatic mutations leading to cancer in the gastrointestinal tract. Although studies to date have not demonstrated this same relationship with lung cancer, we do know that the lungs are subjected to large quantities of ROS under certain conditions as discussed earlier. The formation of mutations from oxidative stress does have other deleterious effects on cells including cellular death by necrosis and apoptosis. Tissue viability is dependent upon mutation correction and replication of the surviving cells to replace those that have died. The ability to enhance cellular survival, after specific oxidative exposures, is evident after increased production of the *hMYH *repair gene in these experiments.

We additionally wanted to determine the level of endogenous expression of the glycosylase repair genes in the pulmonary epithelial A549 cell line. Others have demonstrated how different stressors lead to alterations in the endogenous production of specific repair genes. For example, it has been shown that endogenous gene expression of *hOgg1 *was elevated following exposure to crocidolite asbestos which is known to cause an increase in 8-oxoG levels [48]. It has also previously been reported that treatment of A549 cells with sodium dichromate, a pro-oxidant, leads to a reduction of *hOgg1 *protein expression that was not observed with H_2_O_2 _[49]. One additional study demonstrated a dose dependent down regulation of *hOgg1 *protein expression in rat lung after exposure to cadmium, a known carcinogen associated with the formation of intracellular ROS [50]. In our experiments we were able to demonstrate that both hyperoxia and IR do not appear to impact the endogenous expression of *hOgg1*, *hMYH*, and *hMTH *at 24 and 48 hours following exposure. It was noted that endogenous *hNTH *was reduced after hyperoxia at 24 and 48 hours after the onset of exposure. One would speculate that this reduction in endogenous *hNTH *secondary to hyperoxia is related to either decreased production or increased destruction in response to O_2 _exposure. Over-expression of this repair enzyme did not result in improvements in survival after O_2 _exposure based on our experiments. It may be that endogenous levels are adequate to correct this specific mutational burden for these experiments.

Furthermore, no previous studies have determined how cells over-expressing specific repair genes may impact endogenous expression of the other oxidative BER genes under both normal and oxidative stress conditions. We were also able to demonstrate that endogenous expression of glycosylase repair genes were not altered under these conditions secondary to the over-expression of any of these genes. This is an important finding for interpretation of survival data; protection of cells is due to the over-expression of the specific gene and not due to enhancement of other endogenous repair enzyme levels, at least for the genes studied under these conditions.

Some limitations may exist in using a lung carcinoma cell-line, which likely differs both in proliferative properties as well as in response to oxidative stress in comparison to primary epithelial cells. The enhanced cell growth observed with cell lines may be more reflective of undifferentiated alveolar type II cells which are likely to replace terminally differentiated alveolar type I cells after injury/death due to oxidative stress. This may not be a true reflection of growth under non-toxic conditions when very little cell division is occurring. This is an inherent problem observed when comparing cell lines with primary cells and results need to be interpreted in a way that considers this.

It is difficult to know how this will translate to pulmonary epithelial cells *in vivo *at this stage. It certainly would appear that the protection observed is modest in degree in this pulmonary epithelial cell line. Further experiments assessing the function of the repair enzyme *hMYH *in this model will be important to perform in order to delineate the findings of slowed growth under normal conditions and improved survivability under conditions of O_2 _and IR. More research looking at the potential for combination therapy, including DNA repair mechanisms in conjunction with other antioxidant defense mechanisms may be another approach to enhancing cell survival, which may lead to better clinical outcomes. Alternatively, cell survival may not be the most important end point for hyperoxia studies. Given that 8-oxoG, if left unrepaired, leads to G:C to T:A transversions, there may be an increase in mutational burden by these cells that isn't reflected in cell survival. Further experiments studying the impact on mutation production is underway. Ultimately, experiments need to be done in animal models to determine the translation to *in vivo *pulmonary cells.

## Conclusions

In summary, we have demonstrated that over-expression of the DNA glycosylase repair enzyme *hMYH *may enhance survival of a pulmonary epithelial cell line after exposure to conditions of IR and hyperoxia. We have also demonstrated that over-expression of *hMYH *leads to a slowing of growth of A549 cells under non-toxic conditions, which may in part play a role in this enhancement of survival by providing a wider window of opportunity for repair of oxidized lesions to occur. Lastly, we demonstrated that over-expression does not lead to altered endogenous expression of these repair genes. As the understanding of DNA repair mechanisms continues to grow and the evolution of gene therapy takes place, more treatment options may be available in the clinical setting to help with many disease processes including the damaging effects of oxygen and its metabolites.

## List of abbreviations

apurinic, AP; base excision repair, BER; Dulbecco's modified Eagle's medium, DMEM; deoxyribose phosphate, dRP; enhanced green fluorescent protein, EGFP; fetal bovine serum, FBS; hydrogen peroxide, H_2_O_2_; ionizing radiation, IR; internal ribosomal entry site, IRES; long terminal repeat, LTR; oxygen, O_2_; Sulforhodamine B, SRB; reactive oxygen species, ROS; region of interest, ROI; Tris-Borate-EDTA, TBE; tris-buffered saline Tween-20, TBST; 8-oxoguanine, GO and 8-oxoG

## Authors' contributions

TK conducted the majority of the research experiments, performed the statistical analysis, and drafted the manuscript. MR conducted some of the cell survival experiments and participated in the design of the study. YX and XC helped with production of the lesion specific DNA repair genes. MK conceived of the study, and participated in its design and coordination. All authors read and approved the final manuscript.
